# Effects of Particle Size on the Gas Uptake Kinetics and Physical Properties of Type III Porous Liquids

**DOI:** 10.1021/acsami.3c18998

**Published:** 2024-03-21

**Authors:** Siyuan Liu, Beibei Lai, Stuart L. James

**Affiliations:** School of Chemistry and Chemical Engineering, Queen’s University Belfast, David Keir Building, Stranmillis Road, Belfast BT9 5AG, U.K.

**Keywords:** porous liquid, CO_2_ adsorption, nanometal organic frameworks, aluminum fumarate MOF, size-dependent

## Abstract

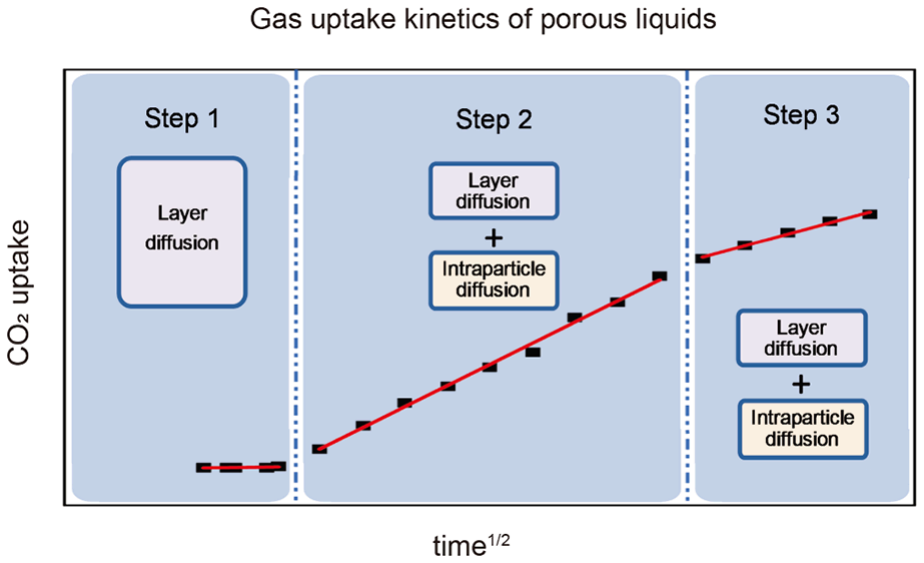

Type III porous liquids (PLs) consist of porous solid particles dispersed in a size-excluded liquid phase and are attracting much attention as novel media for applications such as gas separation. However, the effects of fundamental variables such as particle size on their physical properties are currently largely unknown. Here we study the effects of particle size in a series of porous liquids based on solid Al(OH)(fumarate) (a microporous metal–organic framework, MOF) with particle sizes of 60 nm, 200–600 nm, or 800–1000 dispersed in liquid polydimethylsiloxane (PDMS). Properties examined include physical stability of the dispersion, viscosity, total CO_2_ uptake, and kinetics of CO_2_ uptake. As expected, both physical stability and viscosity decreased with increasing particle size. Unexpectedly, total gravimetric gas uptake also varied with particle size, being greatest for the largest particles, which we ascribe to larger particles having a lower relative content of surface-bound FMA ligands. Various models for the gas uptake kinetic data were considered, specifically adsorption reaction models such as pseudo-first-order, pseudo-second-order, and Elovich models. In contrast to pure PDMS, which showed first-order kinetics, all PLs fit best to the Elovich model confirming that their uptake mechanism is more complex than for a simple liquid. Adsorption diffusion models, specifically Weber and Morris’ intraparticle model and Boyd’s model, were also applied which revealed a three-step process in which a combination of diffusion through a surface layer and intraparticle diffusion were rate-limiting. The rate of gas uptake follows the order PDMS < PL1 < PL2 < PL3, showing that the porous liquids take up gas more rapidly than does PDMS and that this rate increases with particle size. Overall, the study suggests that for high gas uptake and fast uptake kinetics, large particles may be preferred. Also, the fact that large particles resulted in low viscosity may be advantageous in reducing the pumping energy needed in flow separation systems. Therefore, the work suggests that finding ways to stabilize PLs with large particles against phase separation could be advantageous for optimizing the properties of PLs toward applications.

## Introduction

Porous liquids (PLs) are a new type of material that combines fluidity with permanent porosity.^1,2^ They are becoming increasingly studied for a wide range of fields such as gas adsorption^3^ and catalysis.^4^ The combination of fluidity and porosity is attractive because PLs can exhibit selective gas uptake and be applied in continuous flow separation processes, which is not possible for porous solids. Four types of PLs have now been classified.^1^ Type-1 PLs are neat liquids, in which molecular hosts define intrinsic, rigid, and non-self-filling pores. Type-2 PLs are composed of molecular hosts that define a pore and that are dissolved in size-excluded solvents. Type-3 PLs consist of particles of porous framework materials which are dispersed in size-excluded liquid phases. Type-4 PLs are liquid framework structures. Type-3 porous liquids are attractive for applications because of the wide variety of economical porous solid frameworks and liquid carriers from which they can be made. Metal–organic frameworks (MOFs) are one class of porous solid which are important in type-3 PLs because the variability of metal-site and organic ligands gives rise to many possible materials.

Aluminum fumarate (AF) is an important and commercially available^5^ MOF which can be synthesized easily and economically from aluminum salts and fumaric acid (H_2_FMA), using only water as the synthesis solvent, or even under solvent-free conditions by continuous twin screw extrusion.^6^ Because of its high thermal and chemical stability,^7^ AF is being studied for water adsorption and heat transmission.^8^ However, few studies have reported the effect of AF particle size on its properties.

In the study of type-3 PLs, the particle size of the dispersed porous material is a key fundamental variable. However, very little systematic study has been done to date to examine the effects of particle size on the properties of the PL. Rheological properties of UiO-66@PDMS with different particle sizes have been studied by Li et al.^9^ Also, the physical stability of type-3 porous liquids based on porous organic cages with various particle sizes has been investigated by Kai et al.^10^ Herein, the properties of AF and three PLs consisting of AF dispersed in liquid PDMS (polydimethylsilicone) have been studied with AF particles ranging in size from 60 nm to 1 μm.^11−13^ By employing SEM (scanning electron microscopy) and PXRD (powder X-ray diffraction), the particle size and crystallite size have been determined. By combining N_2_-BET (Brunauer–Emmett–Teller) measurements with CO_2_ uptake measurements from 1 to 5 bar, the adsorption capacities and gas uptake kinetics have also been studied. Vibrational viscometry and an optical analytical centrifuge have been used to determine trends in viscosity and physical stability to sedimentation. Also, unusually for PLs, the study provides important basic insight into gas uptake kinetics for and provides some potentially useful pointers regarding optimization of particle size to give PLs with the most desirable properties for use in gas separations.

## Results and Discussion

In a previous study, PLs which consist of AF dispersed in PDMS were studied for CO_2_ uptake measurement because of the high chemical stability of this system.^14^ Herein, three PLs with 12.5 wt % AF dispersed in PDMS (50 cSt) were studied in greater depth.

### AF Porous Solids with Different Particle Sizes

It has been reported that the particle size of AF can be controlled during the synthesis nucleation step by changing the alkalinity of the aqueous synthesis medium.^11^ Higher alkalinity gives faster nucleation resulting in smaller particles. Also, prolonging the reaction time results in larger particles. Based on these observations, AF was synthesized with a range of particle sizes according to literature methods. Specifically, using a short reaction time (1 h) and high alkalinity in water solution (molar ratio of NaOH:H_2_O = 1:60), AF sample AF1 (60–100 nm) was obtained,^11^ while longer reaction time (3 h) and lower alkalinity (molar ratio of NaOH:H_2_O = 1:82) gave sample AF2 (200–600 nm).^12^ By extending the reaction time to 4 d and using dimethylformamide (DMF) as solvent, sample AF3 (800–1000 nm) was obtained.^13^ Key analytical data for samples AF1–3 are given in Table 1. The particle sizes of AF1–3 were determined by SEM (Supporting Information Figures S4–S6). For AF1 this corresponded well to the literature value. For AF2 and AF3, particle sizes have not previously been reported. However, particle sizes increased as expected from AF1 to AF3 from 60 to 100 nm up to 1000 nm. TGA was used to assess the stability and volatile content of AF1–3 (Supporting Information Figure S3). Samples were activated at 150 °C under vacuum for 3 h before analysis (this is reported to be sufficient for the removal of included water^13^). However, for all samples, a 20% weight loss under 100 °C was observed suggesting significant water was present due to rehydration on exposure to ambient air before the measurement. The decomposition temperatures are all between 450 and 500 °C which is consistent with literature.^15^ The crystallite sizes according to PXRD and the Scherrer equation were similar for all samples at 24 ± 2 nm. N_2_-Brunnauer–Emmett–Teller (N_2_-BET) adsorption and desorption measurements were obtained, and the derived surface areas and pore volumes are given in Table 1. Both the specific micropore surface area and pore volume were found to increase systematically with the particle size. We suggest this reflects the decreasing proportion of terminal, surface-bound FMA ligands on the particle surfaces as particle size increases, and thus the total outer surface area of the particles, decreases (Table 1). This hypothesis was supported by metal ICP analysis (Table 1), which showed systematically increasing Al content with increasing particle size. However, we cannot rule out that differences in the number and types of defects in the particle of different sizes also contributes to differences in total gas uptake.

**Table 1:** Key Properties of AF Solid Samples AF1–3

sample	pore surface area (m^2^/g)	pore volume (cm^3^/g)	particle size (nm)	Al content (mg/kg)	crystallite size (nm)
AF1	683.0	0.28	60–100	133	24.2
AF2	747.3	0.30	200–600	143	26.0
AF3	915.6	0.37	800–1000	151	24.2

### AF@PDMS Porous Liquids

#### i Preparation and Chemical Stability

PL1–PL3 were obtained based on solid AF samples AF1–AF3 respectively at 12.5 wt % in PDMS (50 cSt). Preparation involved activated AF solid samples (150 °C, 3 h) being vigorously stirred in the liquid for 24 h to give visually homogeneous dispersions. PXRD patterns of PL1–3 showed in each case sharp peaks due to AF superimposed on a broad hump due to the PDMS component, as expected (Supporting Information Figures S13–S15). This confirms that the AF particles retain their crystallinity upon dispersion.

#### ii Particle Size

The AF particle sizes in PL1–3 were determined by DLS (dynamic light scattering). Results were consistent with the size ranges determined through SEM analyses for AF1 and AF2, specifically 66 nm for PL1 and 237 nm for PL2. For PL3 a broader distribution was seen than for PL1 and PL3 centered at 1468 nm, greater than the particle size range determined by SEM, suggesting a degree of particle aggregation in PL3 (Supporting Information Figure S12).

#### iii Viscosity and Stability to Sedimentation

For nanofluids (i.e., fluids consisting of solid nanoparticles dispersed in the liquid phase), dynamic viscosity is an important factor which will affect properties such as physical stability and thermal conductivity. Currently, there is no generally accepted theory which defines the relationship between a nanofluid’s viscosity and the size of the dispersed particles in a straightforward way. Several recent experimental studies have supported the idea that dynamic viscosity increases with particle size.^16,17^ For example, Nguyen et al. studied alumina-water nanofluids based on various-sized alumina particles and the results indicated that larger particle size resulted in greater viscosity.^18^ Conversely, Lu et al. reported that the viscosity of alumina dispersions in both ethylene glycol and water increased with decreasing particle size.^18^ The same trend was reported by Namburu et al. for silica nanofluids based on ethylene glycol and water.^19^ Further, Prasher et al. found that the viscosity of alumina-polyethylene glycol nanofluids did not vary strongly with particle size.^20^

The dynamic viscosities of PL1–3^21^ were measured at 20 °C using a vibrational viscometer (Table 2). As expected, all of PL1–3 were more viscous than pure PDMS. Also, there was a clear trend that viscosity decreased with increasing particle size. Since PL1–3 have the same loading (12.5 wt %), this trend is consistent with greater numbers of particles and a greater total external surface area resulting in greater interparticle friction.

**Table 2:** Viscosities and Instability Indices of PL1–3

sample	viscosity (mPa·s)	instability index
PL1	407	0.30
PL2	289	0.34
PL3	162	0.35

The stability of the PL dispersions to sedimentation was studied visually as well as with an optical analytical centrifuge (Lumi-Sizer, LUM GmbH, Berlin, Germany) which measures near-infrared (NIR) absorbance as a function of time and position in the sample under centrifugation conditions. Visually, the samples showed no sedimentation during 5 days standing. Under centrifugation conditions equivalent to 11 days at standard gravity, all samples were seen to sediment partially as indicated by greater transmittance at the “top” of the sample over time (Supporting Information Figures S26–S28). The instability index, calculated by dividing the degree of clarification at a given centrifugation time by the maximum clarification,^22^ increased with increasing particle size. This trend is as expected according to previous work^14^ and can be attributed to both the differences in the viscosity^24^ and Stokes’ law which predicts that smaller particles have lower sedimentation terminal velocities.^10,25^

#### iv Gas Uptake

**Figure 1:**
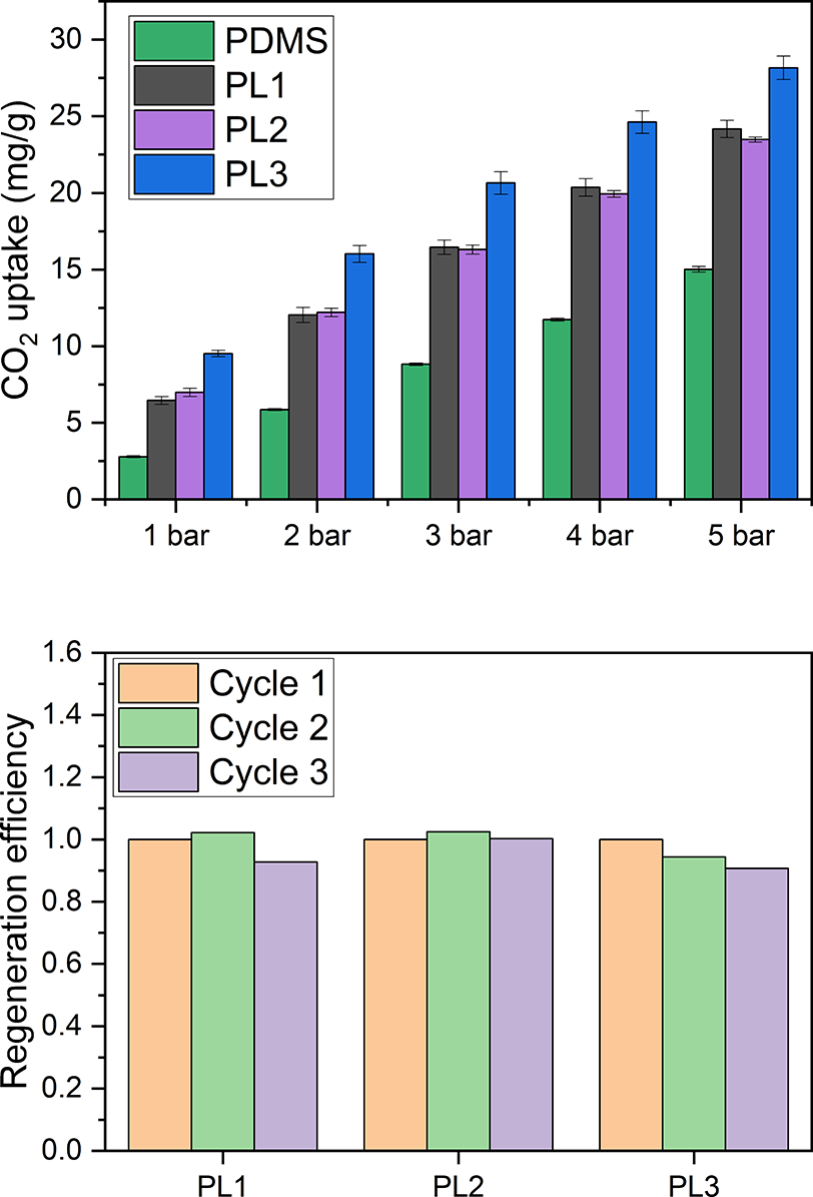
(a) CO_2_ uptake for PDMS and PL1–3 from 1 to 5 bar. As expected, the gas uptakes for PL1–3 all increased with increasing pressure in the range 1–5 bar. (b) Regeneration study of PL1–3 for CO_2_ uptake.

The gas uptake capacities at pressures of 1, 2, 3, 4, and 5 bar and ease of regeneration were studied for PL1–3 using CO_2_ as the probe gas (Figure 1a). The general trend in total CO_2_ uptake for PL1–3 (PL1 ≈ PL2 < PL3) is broadly in line with the trend in the N_2_-BET pore volumes of AF1–3 (0.28, 0.30, and 0.37 cm^3^g^–1^ respectively, Table 1).^26,27^

The regeneration of PL1–3 was studied at 25 °C by loading each porous liquid with CO_2_ at 5 bar and then stirring under reduced pressure (10^–2^ bar) for 2 h, over three cycles. These conditions were generally sufficient to regenerate 90–100% of the PL capacity in each case (Figure 1b) as shown by subsequent uptake measurements, confirming that CO_2_ uptake is reversible, consistent with the physical rather than chemical binding of CO_2_. While 100% regeneration was not demonstrated in all cases, it was sufficient to confirm that substantial regeneration is possible for PL1–3 and the regeneration conditions were not optimized further. Under these conditions, no particular trend in regeneration ability was observed with regard to particle size.

#### v Kinetics of Gas Uptake

Although there are many studies of CO_2_ adsorption kinetics in MOFs,^28,29^ we are unaware of such studies for porous liquids. It was therefore of interest to study in detail the kinetics of gas uptake by these PLs to determine the trends in the rate of adsorbate uptake by PLs and gain insight into the adsorption process. Several adsorption reaction models have been used to identify the rate of adsorbate uptake in both liquids and solids, such as the pseudo-first-order, pseudo-second-order, and Elovich models. Adsorption diffusion models (Weber and Morris’ intraparticle model and Boyd’s model) have also been developed to give mechanistic insight into the adsorption process.

**Figure 2:**
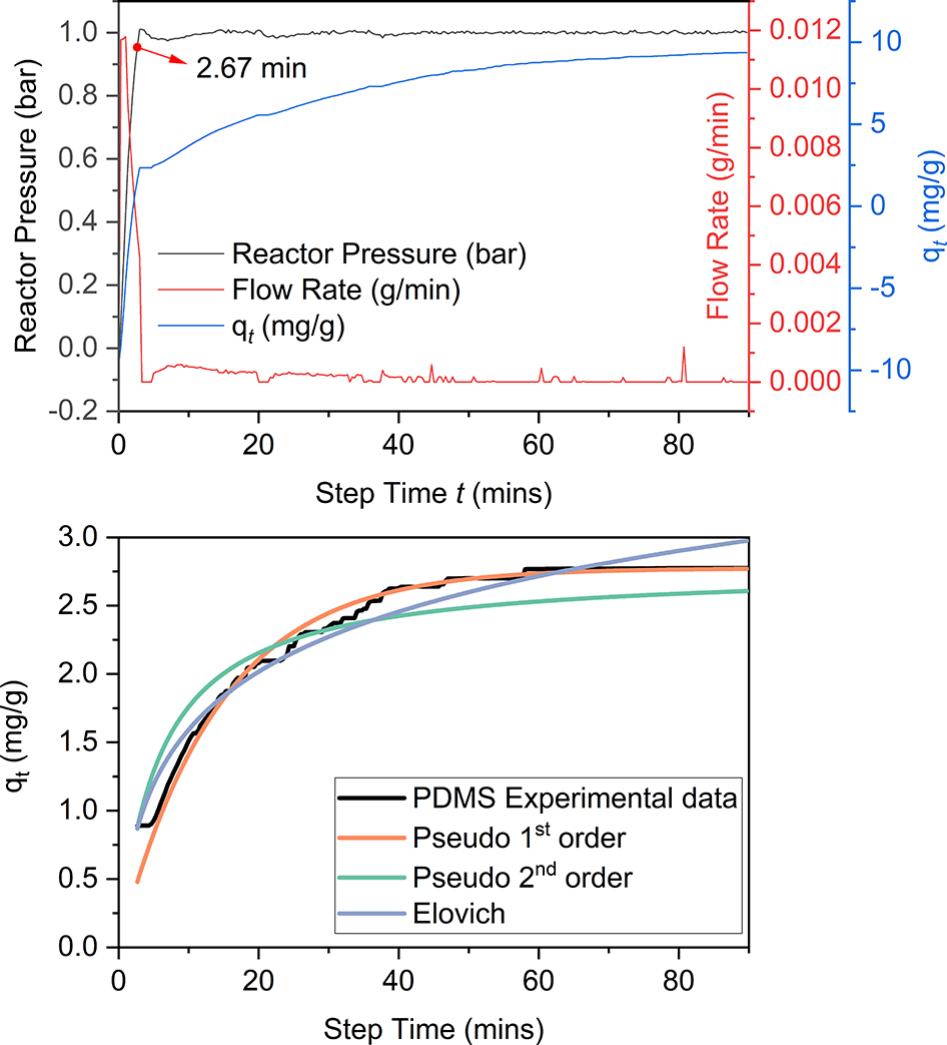
(a) CO_2_ uptake versus time raw data for PL3. (b) CO_2_ uptake versus time for pure PDMS at 298 K, fitted against the three adsorption reaction models. *R*^2^ values are as follows: pseudo first order, 0.9827; pseudo second order, 0.8597; Elovich, 0.9531.

PLs are potentially complex in regard to gas uptake kinetics since uptake occurs initially into the liquid followed by diffusion to, absorption into, and diffusion within the solid particles. PL3 was taken as representative material for this study. A constant-pressure apparatus was used as described in the Supporting Information. Figure 2a shows the CO_2_ uptake raw data versus time (blue), CO_2_ flow rate versus time (red) and reactor pressure versus time (black) of PL3 CO_2_ adsorption at 1 bar. Initially (*t* = 0 min) the chamber is at a pressure of 10^–2^ bar. Gas is then allowed into the chamber to generate a chamber pressure of 1 bar.

This process corresponds to the steep initial slopes of the blue and red lines indicating a rapid increase in chamber pressure and initially fast gas flow, respectively. Since these initial gas flow data do not reflect the uptake of gas into the PL itself, but into the chamber as a whole, they were not used in the kinetic analysis. Specifically, the first 2.67 min of data were not used for kinetic analysis. This initial period during which gas flows rapidly into the chamber is followed by a longer period of slower gas flow as the CO_2_ in the chamber dissolves into the PL, and more gas is consequently allowed into the chamber to maintain the chamber pressure at 1 bar. After *t* = 90 min, the system is approaching equilibrium (i.e., the PL is approaching CO_2_ saturation). Thus, kinetic analysis of gas uptake was done based on data collected for *t* = 2.67 to 90 min.

### Adsorption Reaction Models

To provide a baseline observation, before considering PL1–3, the CO_2_ uptake data for pure PDMS were collected and fitted against the three adsorption reaction models. These adsorption reaction models themselves are described in greater detail below. As shown in Figure 2b, the experimental data only fit well with the pseudo first order model (*R*^2^ = 0.9827).

While Figure 2a shows the raw data only for PL3, the raw data for PL1 and PL2 were similar to this in their general form (Supporting Information Figures S19 and S20). For PL1–3, we initially attempted to fit the experimental data to the three adsorption reaction models summarized as follows.

The pseudo-first-order model was introduced in 1898^30^ by Lagergren and is normally used in fitting gas absorption data during the initial stage (0–40 min)^31,32^ of sorption onto or into both liquids and solids. It is based on the assumption that the rate of sorption is proportional to the number of free sorption sites on or in the sorbent.^33^ This kinetic model is represented by eq 1,
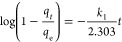
1where *k*_1_ is the kinetic rate constant in the pseudo-first-order model, *q*_*t*_ is the CO_2_ uptake at time “*t*”, and *q*_e_ is the equilibrium CO_2_ uptake at 1 bar.

The pseudo-second-order model, introduced by Ho and McKay in 1998,^30^ is also widely applied to chemical adsorption processes and is described by eq 2,^35^
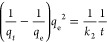
2where *k*_2_ is the kinetic rate constant.

The Elovich model was introduced in 1939.^36^ This model has been used to interpret absorption kinetics both in gaseous and aqueous systems. It is described by eq 3,
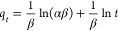
3where α is the rate constant for the initial sorption and β is a constant which is related to the activation energy and the surface coverage.

**Figure 3:**
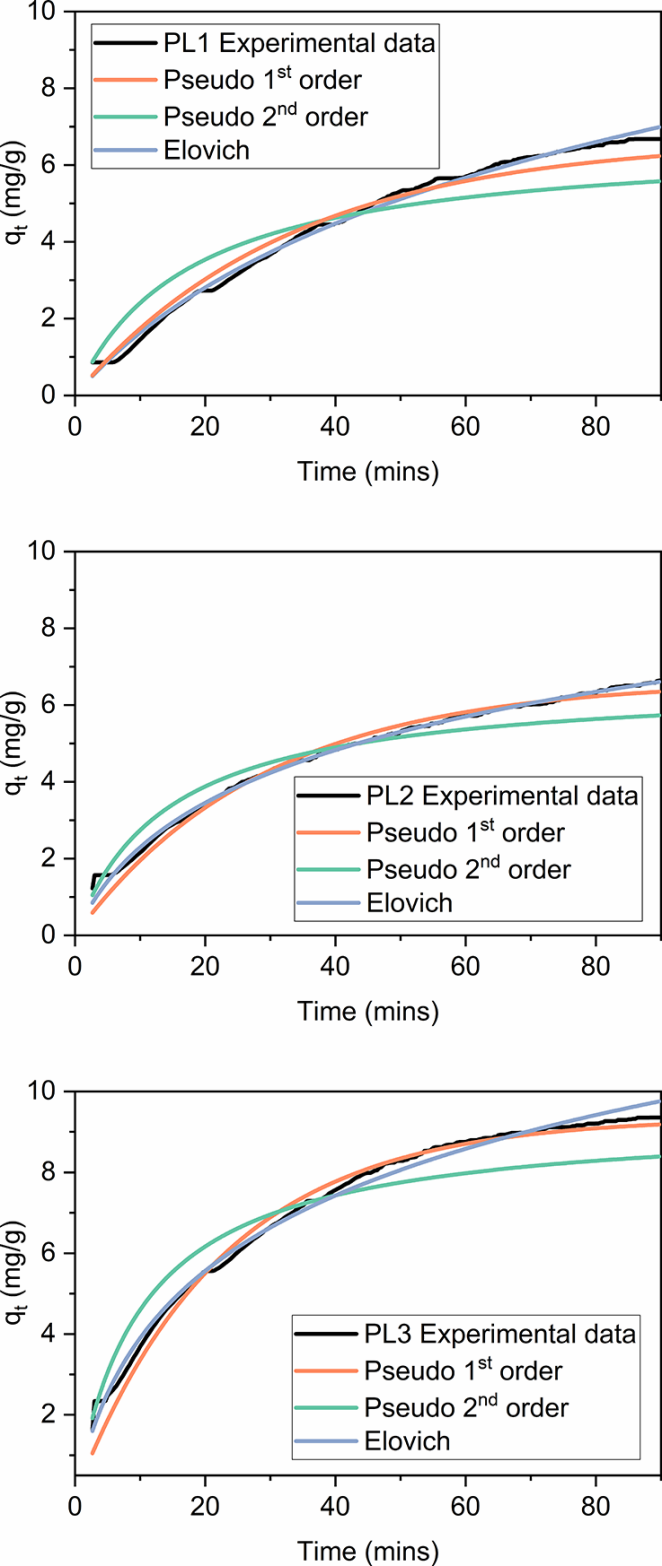
CO_2_ uptake versus *t* for PL1–3 fitted to the three adsorption reaction models at 298 K.

**Figure 4:**
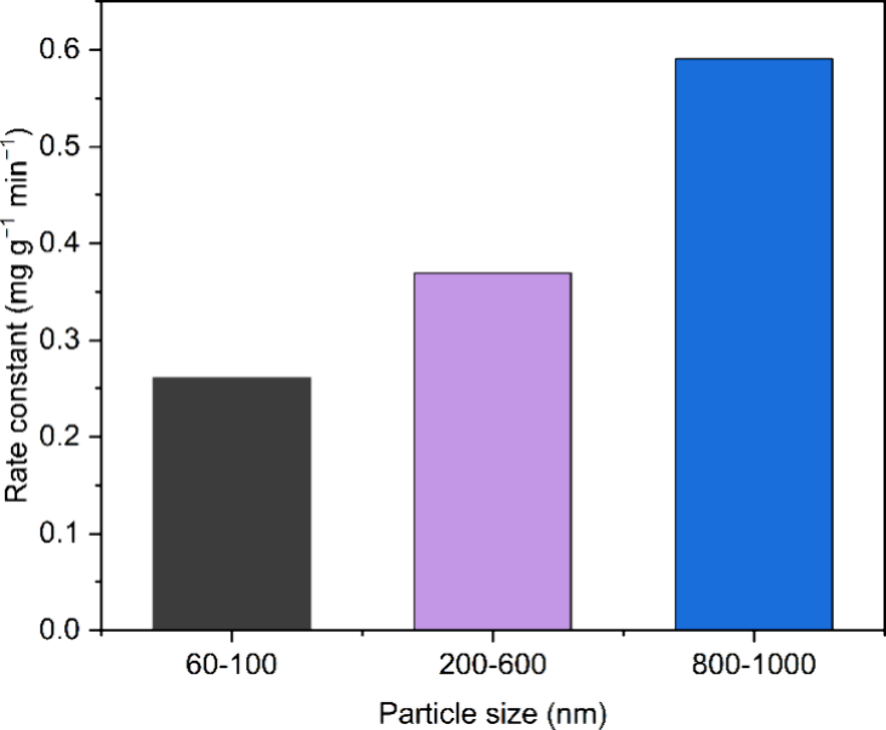
Variation of the Elovich rate constant of adsorption α with particle size in PL1–3.

For PL1–3, in contrast to pure PDMS, pseudo-first-order kinetics were not observed. The Elovich model was found to give the best fit to the experimental data as indicated by the coefficient of determination values (*R*^2^ > 0.99) (Figure 3). We note that PL3 gave a closer match with the pseudo first order model than did PL1 or PL2. However, for PL3, the coefficient of determination for the Elovich model (*R*^2^ = 0.9931) was still greater than for the pseudo-first-order model (*R*^2^ = 0.9908) suggesting that it is the more appropriate interpretation. Overall, for PL1–3 this confirms that the kinetics of gas uptake into the PLs are more complex than into a pure liquid, as anticipated. An interpretation of the Elovich model is that it relates to heterogeneous systems in which not all binding sites are identical.^37^ As such it seems appropriate to PLs in which binding can occur in the liquid and/or solid particles. It was noted that the rate constant α generally increased with the AF particle size in PL1–3 (Figure 4). Relevant parameters are given in Table 3. The trend in the rate of gas uptake is discussed below, following further kinetic analysis.

**Table 3:** Adsorption Reaction Model Parameters for the Elovich CO_2_ Uptake into PL1–3

	Elovich parameters		
	α	β	*R*^2^
PL1	0.261	0.320	0.9950
PL2	0.369	0.364	0.9950
PL3	0.591	0.276	0.9931

### Adsorption Diffusion Models

Although the Elovich model is useful to describe the overall adsorption behavior of PL1–3, it gives no mechanistic interpretation regarding the various potential rate-limiting steps in the adsorption process. In particular, CO_2_ uptake into a PL can be considered to occur in 4 stages as follows (Figure 6).^38,39^ (1) CO_2_ molecules enter PDMS and diffuse through the liquid (bulk diffusion). (2) CO_2_ molecules pass through a liquid boundary layer between the AF surface and the bulk liquid to arrive at the surface of the AF particles (layer diffusion), and simultaneously, some CO_2_ molecules remain in the PDMS. The boundary layer is the solid–liquid interface, and the CO_2_ concentration is greater on the side of the interface closer to the PDMS than on the side closer to the AF surface. (3) CO_2_ molecules which have arrived at the surface of the AF particles, pass into the particle, diffusing through the porous structure (intraparticle diffusion) until they arrive at favorable binding sites in the pores. (4) Finally, binding occurs on the binding sites in the solid particles. Because PLs are rapidly stirred during the experiment, the first stage can be assumed to be fast and so will not limit the adsorption rate. In particular, our experiments were conducted at 500 rpm. Results obtained at 1400 rpm were closely comparable and so we conclude that 500 rpm is fast enough to render bulk diffusion nonrate determining. Stage 4 is also fast since the binding is merely physical rather than chemical, and so will not limit the rate. Therefore, the rate can largely be expected to be limited by stage 2 or 3, or a combination of both. Adsorption diffusion models (Weber and Morris intraparticle model and Boyd’s model) were applied to gain insight into these potential rate-limiting steps in this adsorption process.

**Figure 5:**
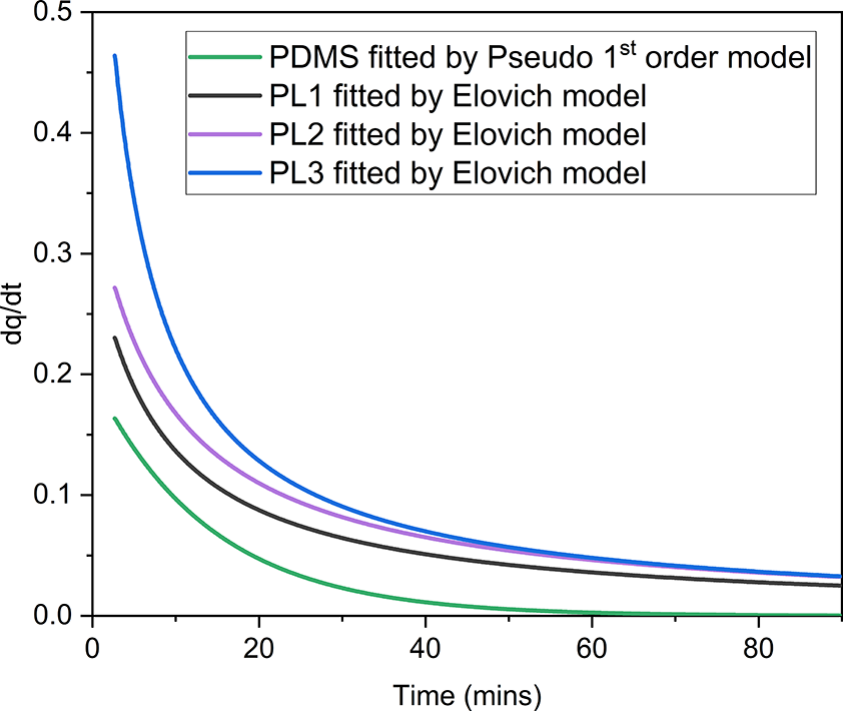
First differential of CO_2_ uptake vs time for PDMS and PL1–3 fitted data, PDMS with pseudo-first-order fitting PL1-3 with Elovich fitting.

**Figure 6:**
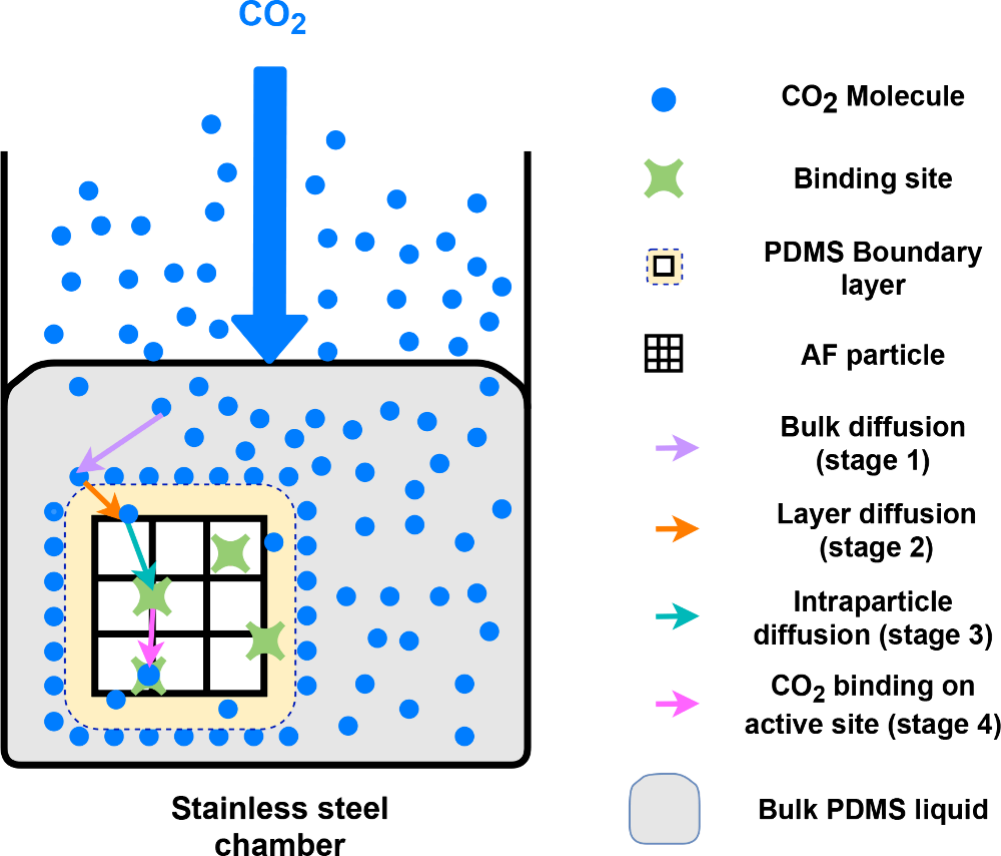
Schematic diagram to illustrate the four stages of gas uptake into PL1–3.

**Figure 7:**
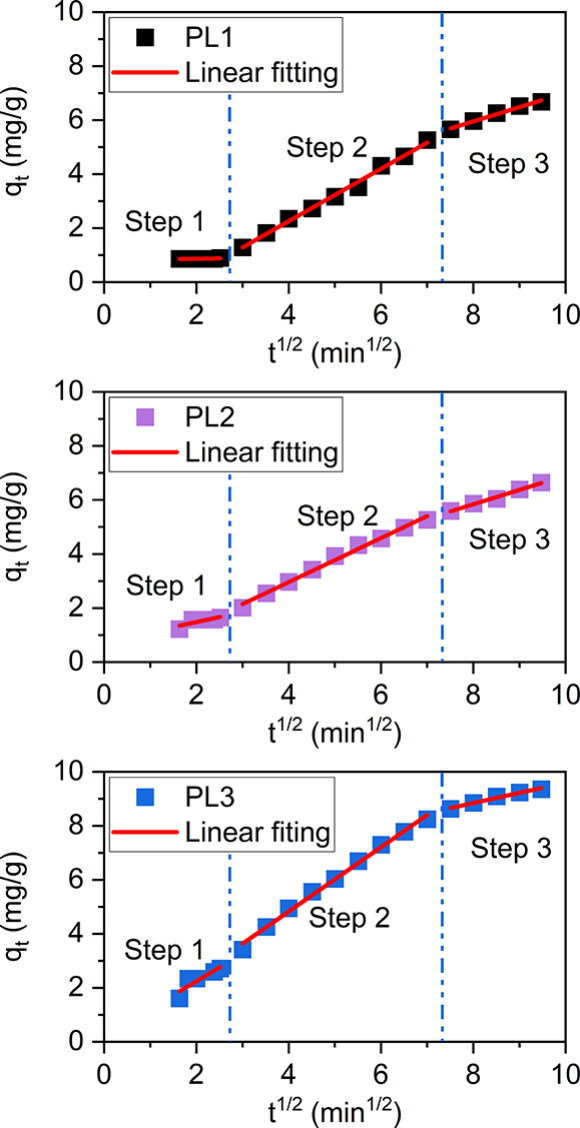
CO_2_ uptake versus *t*^1/2^ of PL1–3 to show fitting to the Weber and Morris intraparticle models at 298 K.

The Weber and Morris intraparticle model was introduced in 1963 and has been successfully applied to pollutant adsorption from wastewater onto active carbon. It is useful for elucidating the role of intraparticle diffusion (stage 3) in the adsorption process,^40^ and is described by eq 4,
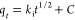
4where *q*_*t*_ is the uptake at time *t*, *k*_i_ is the intraparticle diffusion rate constant, and *C* is a constant. If intraparticle diffusion is important in determining the rate in the adsorption process, plots of *q*_*t*_ vs *t*^1/2^ are linear. If intraparticle diffusion is the rate-limiting step, the intercept of the plot is zero. If there are other rate-limiting steps, the intercept is nonzero. Figure 7 shows plots of *q*_*t*_ vs *t*^1/2^ for PL1–3. Each plot has been interpreted as having three steps. A similar 3-step interpretation was reported previously in CO_2_ adsorption of mesoporous silica MCM-4.^41,42^ In step 1, the data are scattered and nonlinear, which indicates that during this period, intraparticle diffusion is not rate-limiting. This is intuitively reasonable since during this early period gas molecules may not yet have entered the AF particles. Therefore, the rate-limiting process in step 1 would appear to be layer diffusion. In steps 2 and 3, the plots are linear (Table 4) and do not pass directly through the origin, which indicates that during these two periods, intraparticle diffusion is rate-limiting but is not the only rate-limiting step. Therefore, both layer diffusion and intraparticle diffusion appear to be rate-limiting during these steps.

**Table 4:** Weber and Morris Intraparticle Model Parameters (Average) for the CO_2_ Uptake into PL1–3

		PL1	PL2	PL3
Step 1	Intercept	0.812	0.744	0.196
	Slope	0.027	0.369	1.025
	*R*^2^	0.4472	0.6361	0.7860
Step 2	Intercept	–1.620	–0.291	0.064
	Slope	0.969	0.813	0.9947
	*R*^2^	0.9943	0.9915	0.9947
Step 3	Intercept	1.730	1.577	5.840
	Slope	0.528	0.533	0.376
	*R*^2^	0.9901	0.9916	0.9819

Boyd’s model is used to determine whether the rate-limiting step is layer diffusion (stage 2). This model is represented by eq 5,
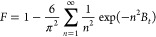
5where *F* is the fractional uptake at time *t* (*F* = *q*_*t*_/*q*_e_), and *B*_*t*_ is a function of *F* and is represented as eq 6,
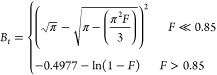
6

**Figure 8:**
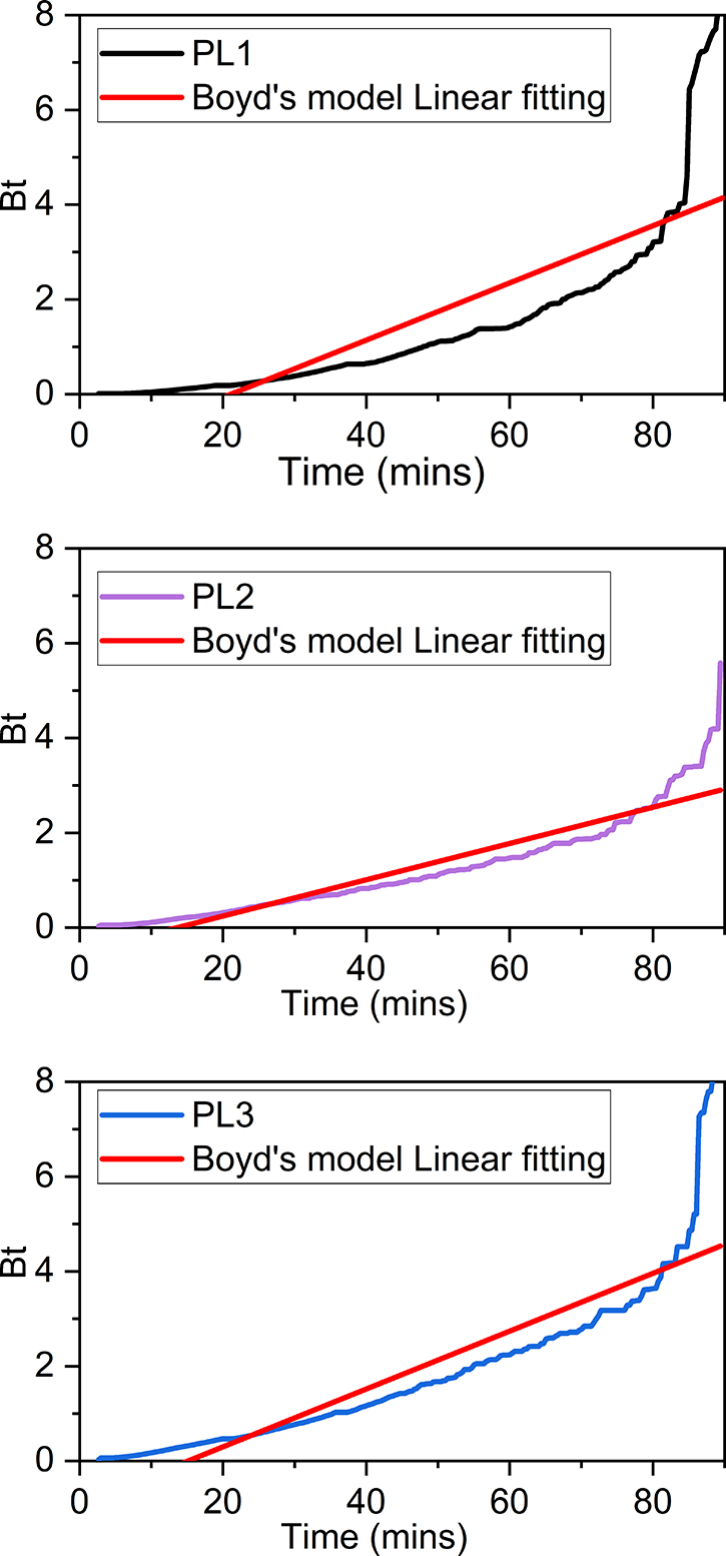
CO_2_ adsorption experiment of PL1–3 fitting with Boyd’s models at 298 K.

If a plot of *B*_*t*_ vs *t* is nonlinear or is linear but does not pass through the origin, layer diffusion is considered play an important role in the adsorption process (i.e., layer diffusion is a rate-limiting factor).^43^ As shown in Figure 8 and Table 5, Boyd’s model plots of PL1–3 are all nonlinear, which indicates that layer diffusion is indeed rate-limiting over the whole adsorption process, which is consistent with the results from the Weber and Morris intraparticle model.

**Table 5:** Boyd’s Model Parameters for the CO_2_ Uptake into PL1–3

	*R*^2^ in Boyd’s model
PL1	0.91854
PL2	0.87356
PL3	0.79175

**Figure 9:**
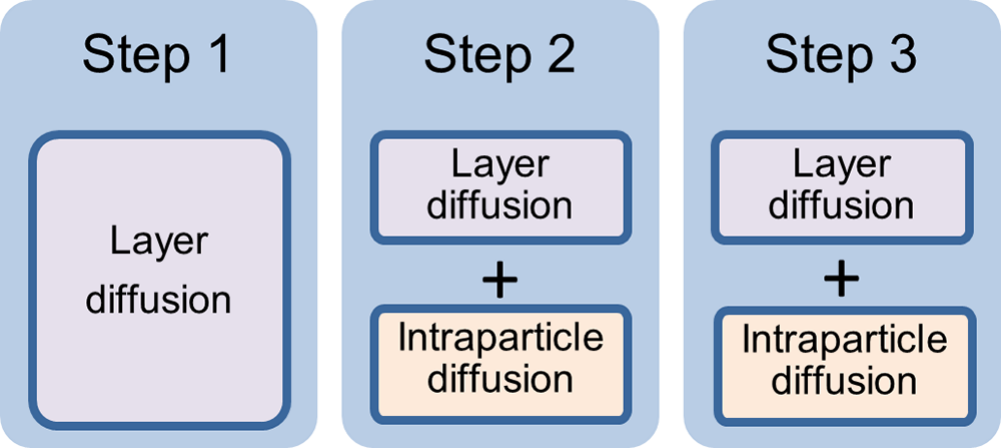
Rate-limiting processes during CO_2_ uptake into PL1–3.

In conclusion, this kinetic study is consistent with the following interpretation. The kinetics of gas uptake can be described as occurring in three steps (Figure 9). Initially, in step 1, layer diffusion is rate-limiting. In steps 2 and 3, both layer diffusion and intraparticle diffusion are both rate-limiting. Step 1 may correspond to the period before which the gas molecules have entered the AF particles, and steps 2 and 3 correspond to the period during which gas molecules have entered these AF particles. However, currently, it remains unclear how to physically interpret the transition from step 2 to step 3.

With regard to the observed trend in the rate of gas uptake noted above (Figure 5, PDMS < PL1 < PL2 < PL3) we note the following points. First, to test the conclusion from the kinetic analysis that bulk diffusion is not rate-determining, it could be interesting to compare the initial rates of gas uptake into PDMS and PL1–3 since at that time only gas diffusion into the bulk liquid phase would be occurring, i.e. layer diffusion and intraparticle diffusion would not yet be taking place. However, this is not possible because the initial uptake data (0–2.67 min) could not be used in the analysis as this period also substantially involves filling of the chamber head space, as explained above. Second, it would be interesting to try to interpret the observed rate trend in terms of the rate-determining processes identified in the kinetic analysis, specifically layer diffusion and intraparticle diffusion. However, mass transfer mechanisms into and within microporous materials are complex and not always well-understood.^44^ For example, the presence of defects can have profound effects on gas diffusion into and within microporous particles, and although more defects might be expected in the smaller particles, the concentration and types of defects which may be present are not known in the current work. With regard to layer diffusion, a further complication is that we currently have no knowledge of the structure, dynamics, and depth of the boundary layer at the PDMS-AF interface or how it might vary with particle size (although we note with interest the work of Sheng et al.^45^ on type 3 porous ionic liquids in which TEM analysis revealed a substantial ∼100–200 nm adsorbed layer of IL at the MOF-IL interface and it is possible that a similar boundary layer exists in PL1–3). Overall, therefore, while we note the clear systematic trend in rate of gas uptake, a mechanistic interpretation of this trend is not possible at this time. It would be valuable to study this aspect in the future however.

## Conclusions

In summary, we have developed AF-based type-3 PLs with different particle sizes and characterized both the solid AF samples and their corresponding porous liquids to investigate the relationship between particle size and the properties of the resulting PLs. Trends in viscosity, stability to sedimentation, CO_2_ uptake capacity, and kinetics have been studied. The increase of AF particle size results in not only a decrease of viscosity and physical stability but also an increase in gravimetric CO_2_ uptake. The kinetics study showed that, in contrast to simple first order kinetics seen for CO_2_ uptake in PDMS, the Elovich model gave the best fit to the uptake data for PL1–3, consistent with the presence of more than one type of binding site (i.e., PDMS and AF). All PLs exhibited faster gas uptake than did PDMS, and the rate of uptake increased with AF particle size. Adsorption diffusion models were consistent with there being three diffusion steps in the CO_2_ uptake of PL1–3. In step 1, layer diffusion provides the main resistance to mass transfer. In steps 2 and 3, both layer diffusion and intraparticle diffusion resist mass transfer. The difference between steps 2 and 3 lies in their distinct rate constants. The scenario in which both layer diffusion and intraparticle diffusion collectively govern the adsorption rate aligns with the findings reported by Ding and co-workers on Alfum pellets with a cellulose binder.^46^

Overall, one pointer that arises from this study is that for PLs with high gravimetric gas uptake and fast uptake kinetics, large particles may be preferred. Also, the fact that large particles resulted in low viscosity may be advantageous in reducing the pumping energy needed in flow separation systems. However, large particles are also more prone to sedimentation. Therefore, finding ways to stabilize PLs with large particles could be advantageous for optimizing the properties of PLs toward gas separation applications.

## Experimental Section

### Materials and Measurements

Aluminum sulfate octadecahydrate (Al_2_(SO_4_)_3_·18H_2_O), fumaric acid (C_4_H_4_O_4_), sodium hydroxide (NaOH), dimethylformamide (DMF)(C_3_H_7_NO), methanol (CH_3_OH), and polydimethylsiloxane (PDMS, 50 cSt) were purchased from Sigma-Aldrich, and aluminum chloride hexahydrate (AlCl_3_·6H_2_O) was bought from Alfa-Aesar. SEM images were obtained with a FEI Quanta FEG-Environmental scanning electron microscope. PXRD measurements were recorded on a PANanalytical X’Pert Pro X-ray diffractometer with Cu as the X-ray source (1.5405 Å). All samples were measured ex situ using a spinning stage, from 2Θ = 5–50° with a step size of 0.0167°. Thermogravimetric analysis (TGA) and inductively coupled plasma (ICP) were measured by the Analytical Service Environmental Protection (ASEP) unit of the School of Chemistry and Chemical Engineering. A NOVA 4200e BET machine was employed for the N_2_-BET measurement. Dynamic viscosity was measured with an AND SV-1A Vibro-Viscometer. 2 mL of porous liquid was placed in the cuvette of the viscometer with temperature maintained at 20 °C by a water bath. Dynamic light scattering (DLS) data of PL1–3 were measured with a Malvern NanoZS ZEN3500 instrument at 25 °C. Transmitted light was collected every five seconds. CO_2_ uptake measurements were analyzed using a Parr reactor based on mass flow (Figure S17). High-pressure gas uptake measurements were conducted using barometric apparatus with controlled pressure, temperature and stirring speed. The amount of adsorbed gas was measured using a mass flow controller (Brooks GF80 with accuracy is <1% SP). 30 g of PL1(1–3) was placed in the sample cell, stirred at 500 rpm, and subjected to vacuum for 2 h. The gas was then allowed to enter the sample cell until the pressure was 1 bar, and the gas flow then controlled to maintain a pressure of 1 bar using a mass flow controller which recorded the amount of gas intake gas every 0.333 s. The adsorption process lasted 90 min.

### Synthesis of AF1–3

#### Synthesis of AF1

Aluminum sulfate octadecahydrate (19.98 g, 30 mmol) was dissolved in distilled–deionized water (75 mL) to form solution A. Fumaric acid (6.96 g, 60 mmol) and sodium hydroxide (6.00 g, 150 mmol) were mixed with distilled–deionized water (87 mL) to form solution B. Solution B was added dropwise into solution A with stirring. The mixture was stirred at 90 °C for 1 h. The obtained white solid precipitate was collected by centrifugation and washed with methanol (3 × 100 mL) before being dried in air. The powder was activated at 150 °C for 3 h.

#### Synthesis of AF2

Aluminum sulfate octadecahydrate (13.80 g, 20 mmol) was dissolved in distilled–deionized water (60 mL) and stirred at 60 °C for 1 h to form solution C. Fumaric acid (4.8 g, 41 mmol) and sodium hydroxide (3.59 g 90 mmol) were mixed with distilled–deionized water (72 mL) to form solution D. Solution D was added dropwise into solution C carefully to form a clear solution. The mixture was stirred at 60 °C for 2 h. The obtained white solid precipitate was collected by centrifugation and washed with methanol (3 × 100 mL) before being dried in air. The powder was activated at 150 °C for 3 h.

#### Synthesis of AF3

Aluminum chloride hexahydrate (7.35 g, 30 mmol) and fumaric acid (4.20 g, 36 mmol) were dissolved in dimethylformamide (150 mL). The mixture was stirred at 130 °C for 96 h. The obtained white solid precipitate was collected by centrifugation and washed with methanol (3 × 100 mL) before being dried in air. The powder was activated at 150 °C for 3 h.

### Synthesis of PL1–3

For synthesis of 12.5 wt % porous liquids, aluminum fumarate MOFs (AF1, AF2, or AF3, 4 g) was activated at 150 °C and vigorously stirred with polydimethylsiloxane (50 cSt) (28 g) (600 rpm) overnight to form visually homogeneous dispersions PL1, PL2, or PL3.

## Supporting Information Available

Synthesis, characterization figure, CO_2_ adsorption capacity, and kinetic fitting data (PDF)

## Abbreviations

MOF: metal–organic framework
AF: aluminum fumarate
PL: porous liquid
PDMS: polydimethylsiloxane (50 cSt)
FMA: fumaric acid
NIR: near-infrared
AF1–3: aluminum fumarate samples 1–3
PL1–3: porous liquids 1–3
