# Supplementary material for: Effects of Particle Size on the Gas Uptake Kinetics and Physical Properties of Type III Porous Liquids
Source: ACS Appl Mater Interfaces. 2024 Mar 21;16(13):16436–44. doi: 10.1021/acsami.3c18998 (PMC10995940; doi:10.1021/acsami.3c18998)
Supplement: Supplementary file 1 — am3c18998_si_001.pdf [file am3c18998_si_001.pdf]

Supporting information

Effects of particle size on the gas uptake kinetics and physical properties of type III porous liquids

Siyuan Liu, † Beibei Lai, † and Stuart L. James* †

†School of Chemistry and Chemical Engineering, Queen's University Belfast, David Keir Building, Stranmillis Road, Belfast, BT9 5AG, UK.

*Corresponding author: S.James@qub.ac.uk

Table of contents

1. Characterization of Aluminum fumarate MOFs 2
2. Characterization of Type-3 porous liquids 10
3. CO_2 uptake measurement 14
   3.1 CO_2 uptake capacity measurement 14
   3.2 CO_2 uptake kinetics raw data of PL1-2 14
4. Regeneration study 16
   4.1 Chemical stability 17
   4.2 Sedimentation stability 19

1. Characterization of Aluminum fumarate MOFs

Aluminum fumarate MOFs were characterized by PXRD, IR, TGA, Metal analysis (ICP), SEM, and BET for T-plot micropore areas and pore volumes.

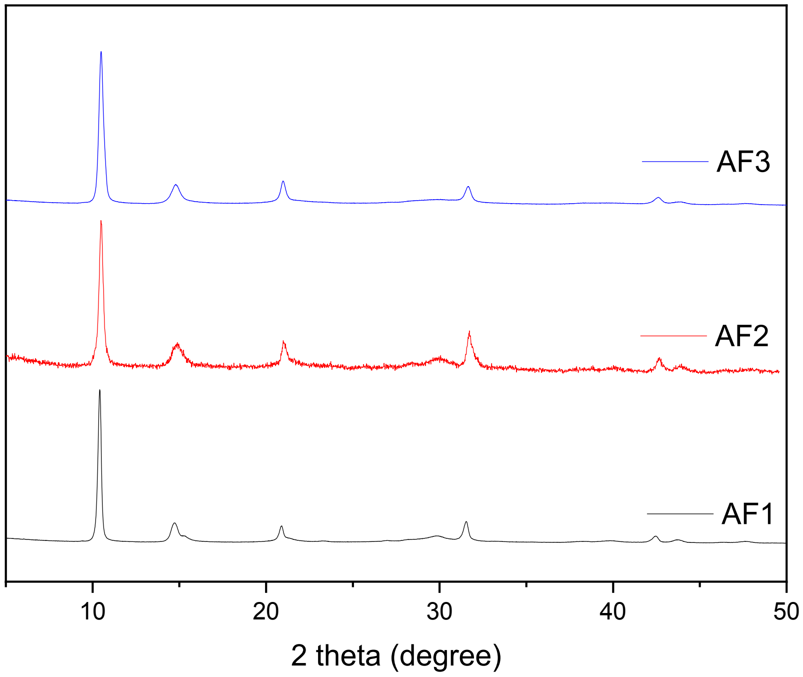

Figure S1. PXRD pattern of porous solids AF1-3.

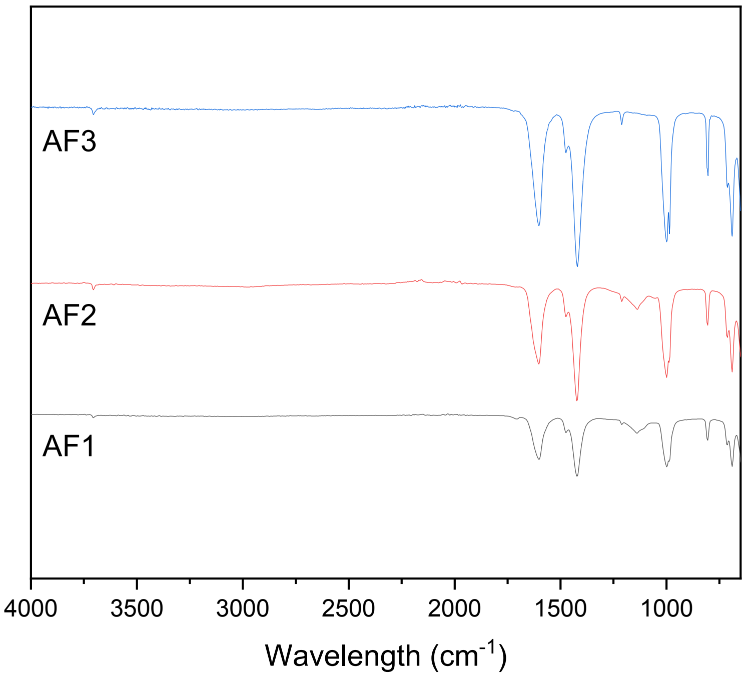

Figure S2. IR spectrum of porous solids AF1-3.

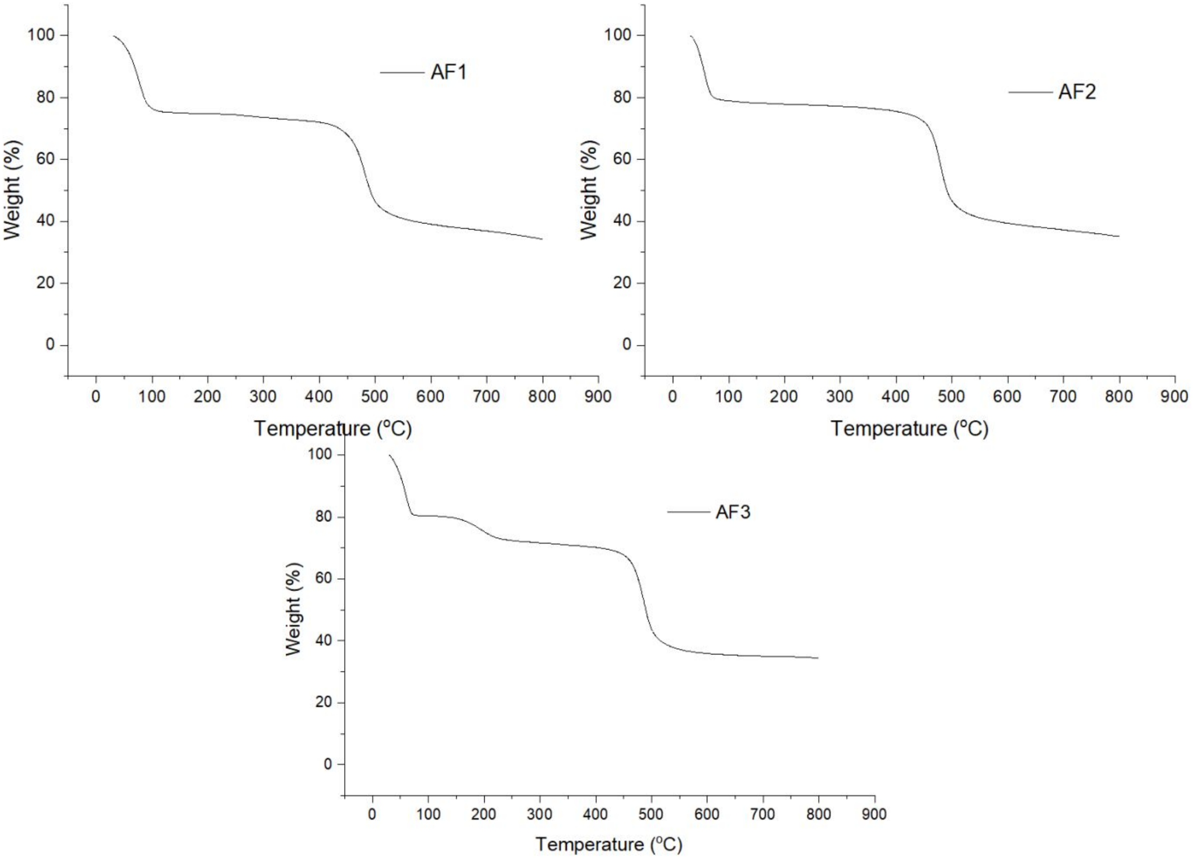

Figure S3. TGA curves of AF1-3.

Table S1. Metal analysis of porous solids AF1-3

Sample	Al content, mg/kg
AF1	132860.4
AF2	143148.6
AF3	151147.6

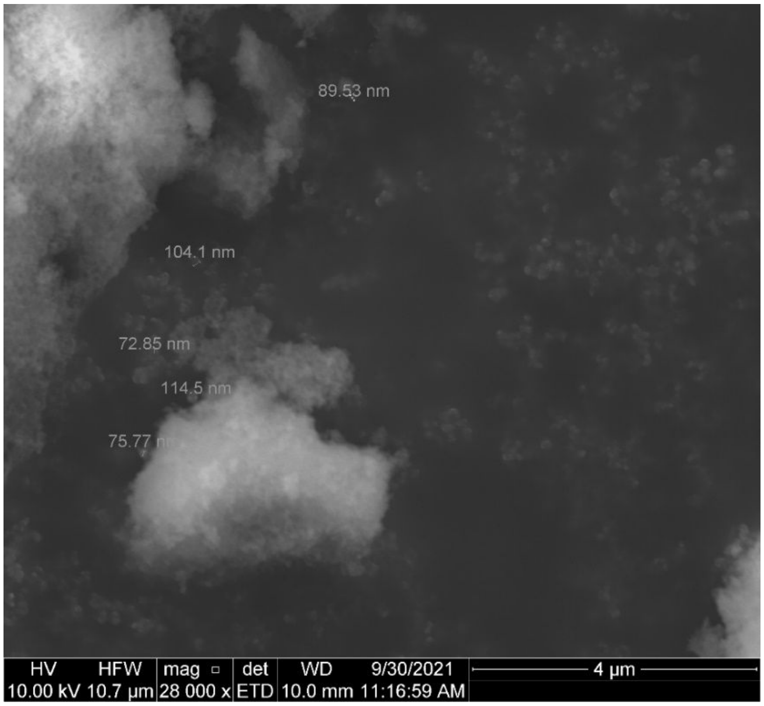

Figure S4. SEM image of AF1.

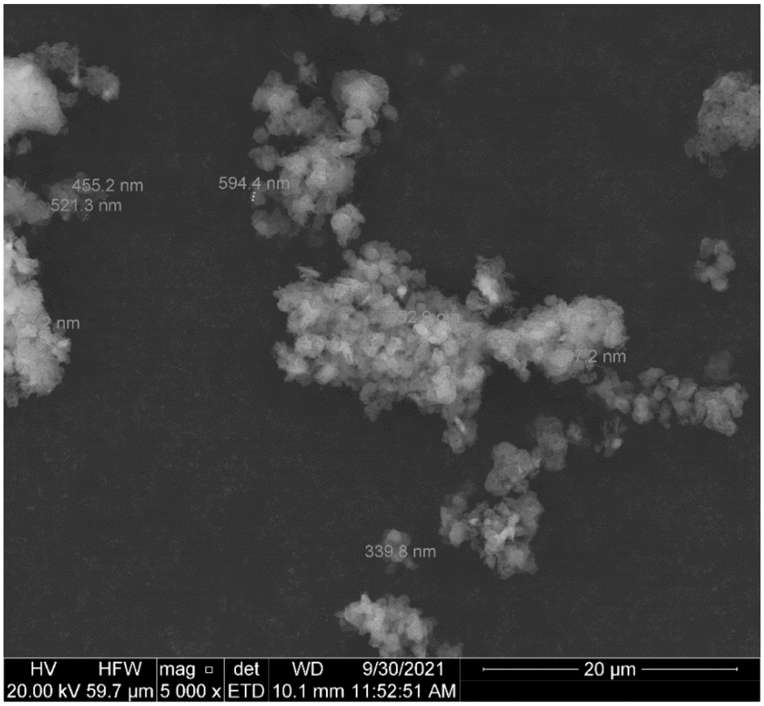

Figure S5. SEM image of AF2.

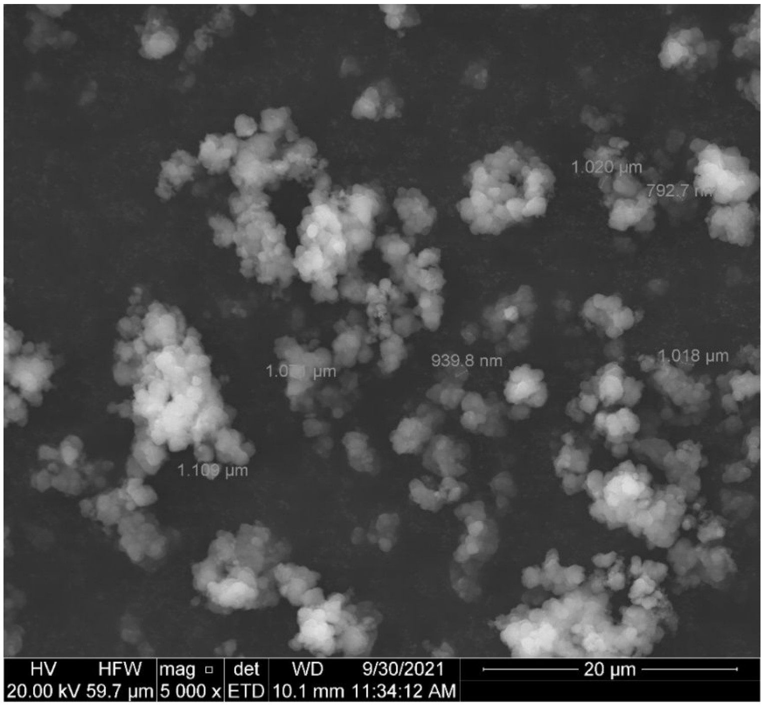

Figure S6. SEM image of AF3.

The N_2-BET adsorption-desorption measurements were measured at 77 K. The corresponding micropore areas and pore volumes were obtained from the BET data by the T-plot method.

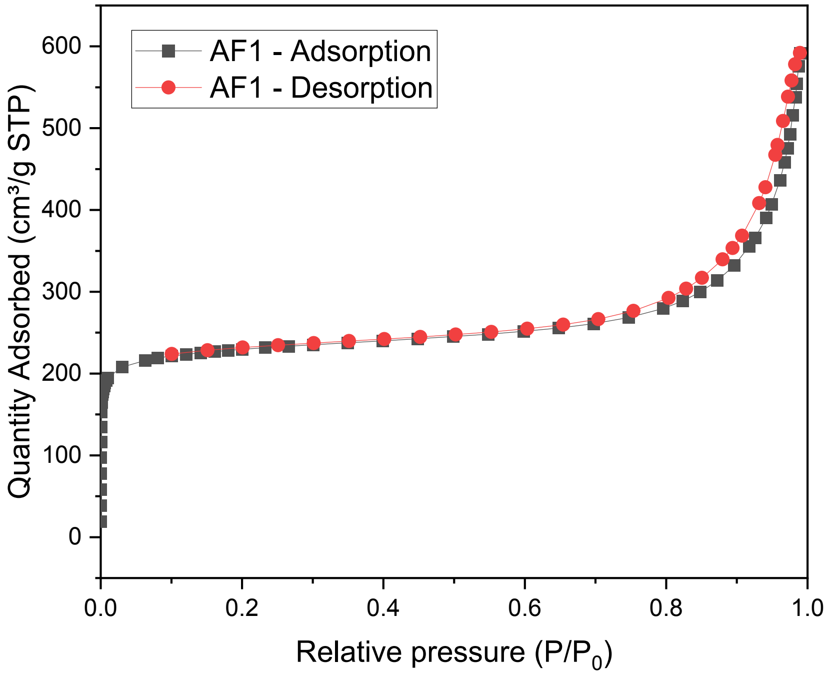

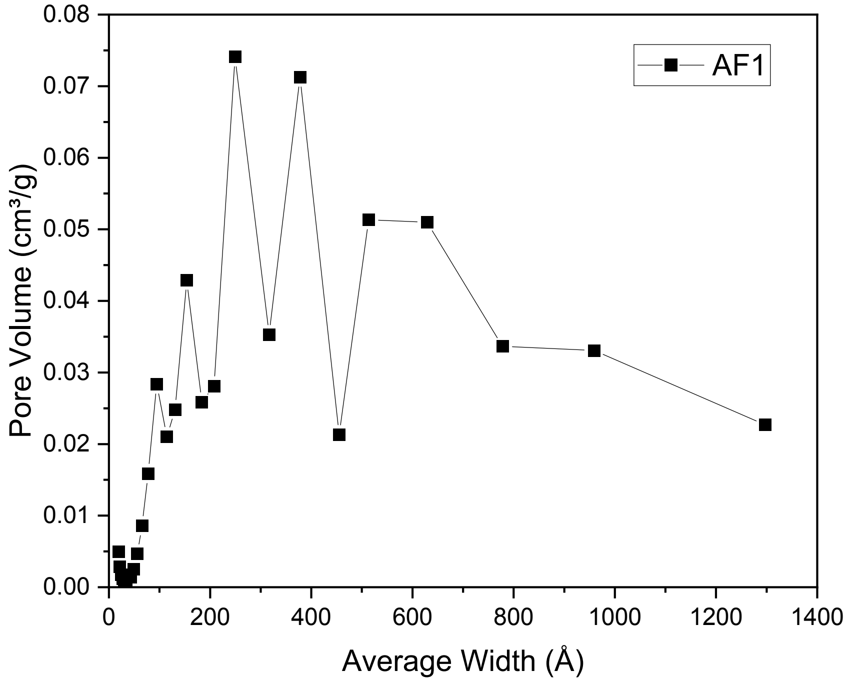

Figure S7. BET isotherms and BJH pore size distribution of AF1.

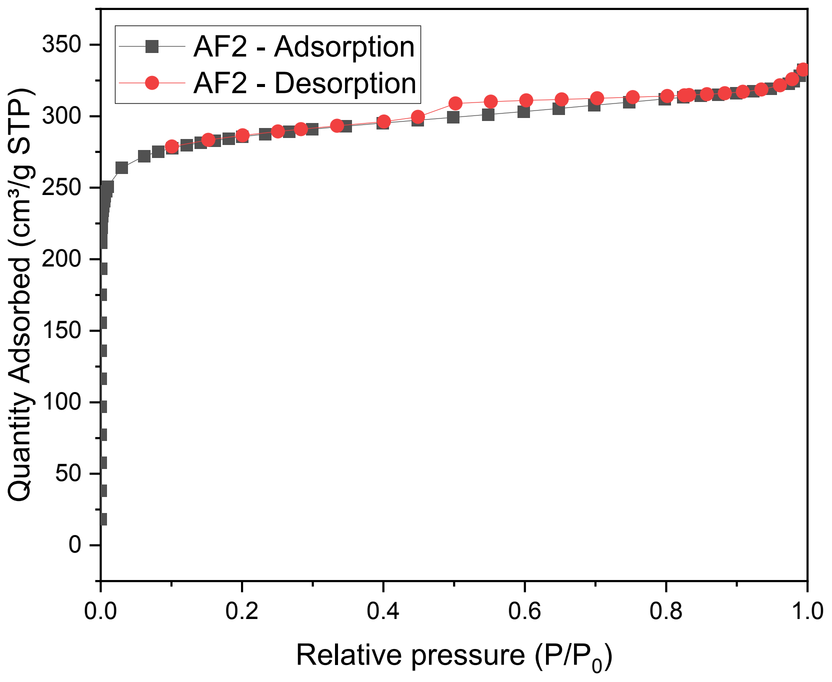

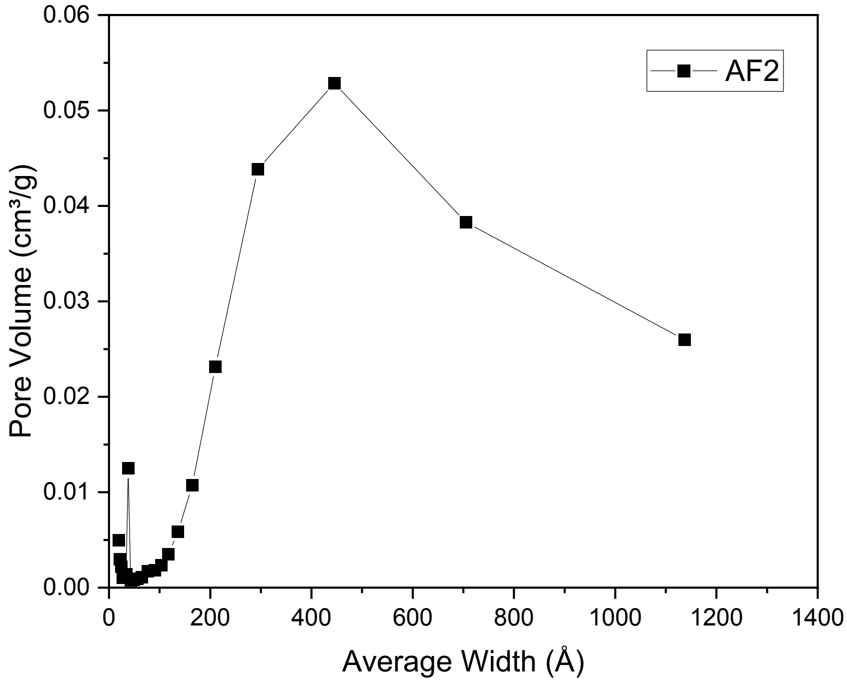

Figure S8. BET isotherms and BJH pore size distribution of AF2.

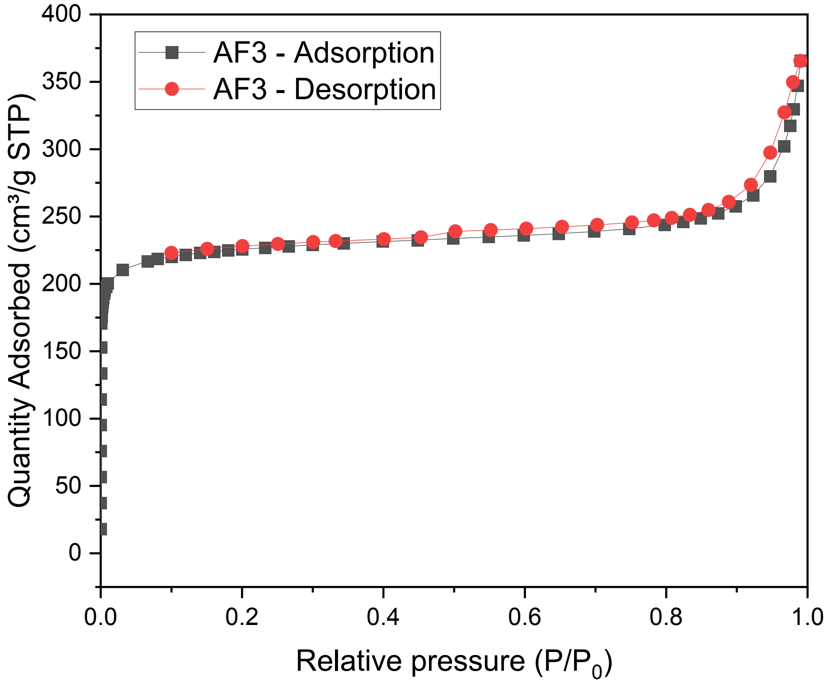

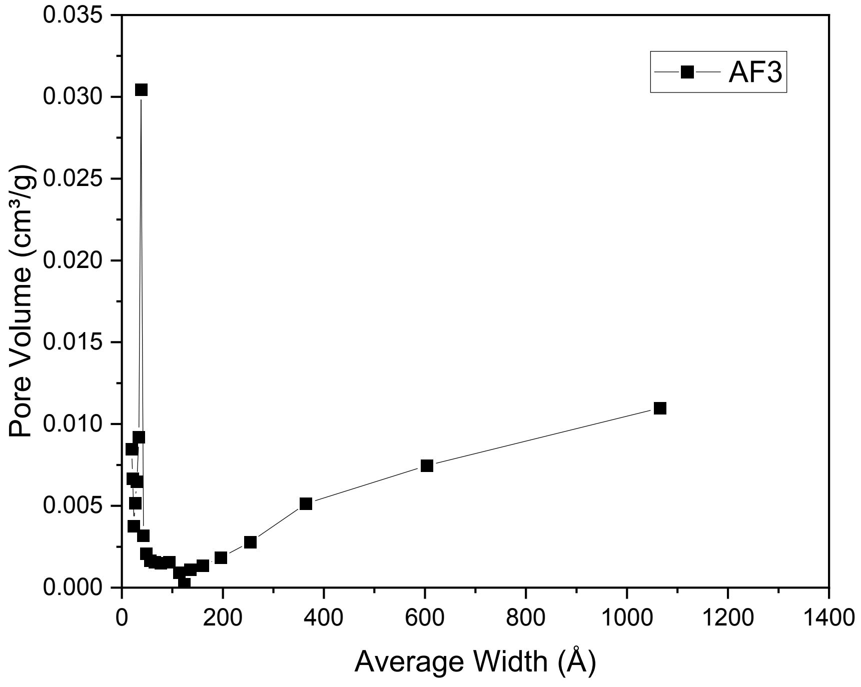

Figure S9. BET isotherms and BJH pore size distribution of AF3.

2. Characterization of Type-3 porous liquids

Type-3 porous liquids were characterized by DLS, PXRD, and vibrational viscometer.

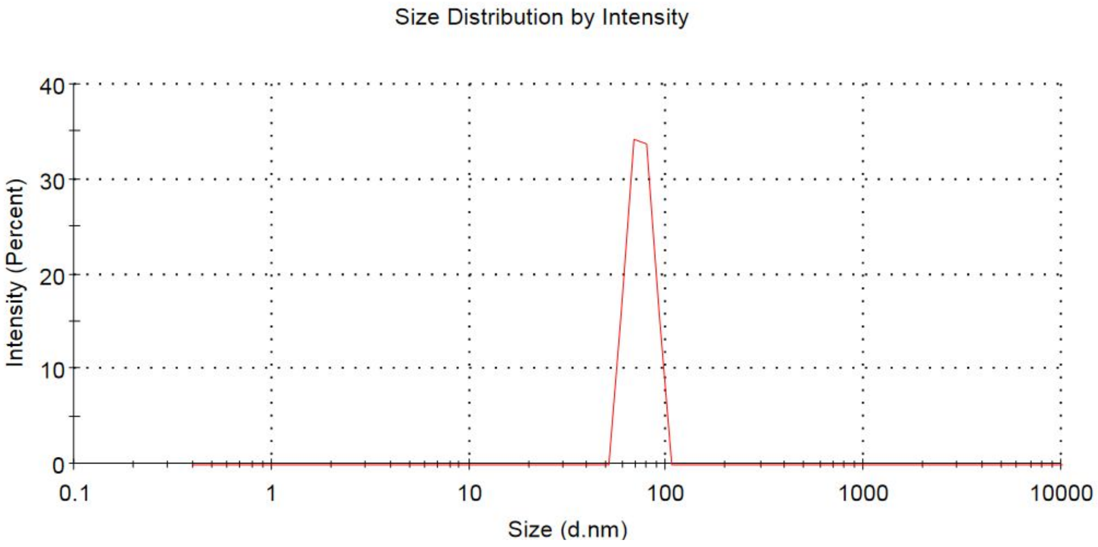

Figure S10. DLS report of AF1 (Z average: 66 nm).

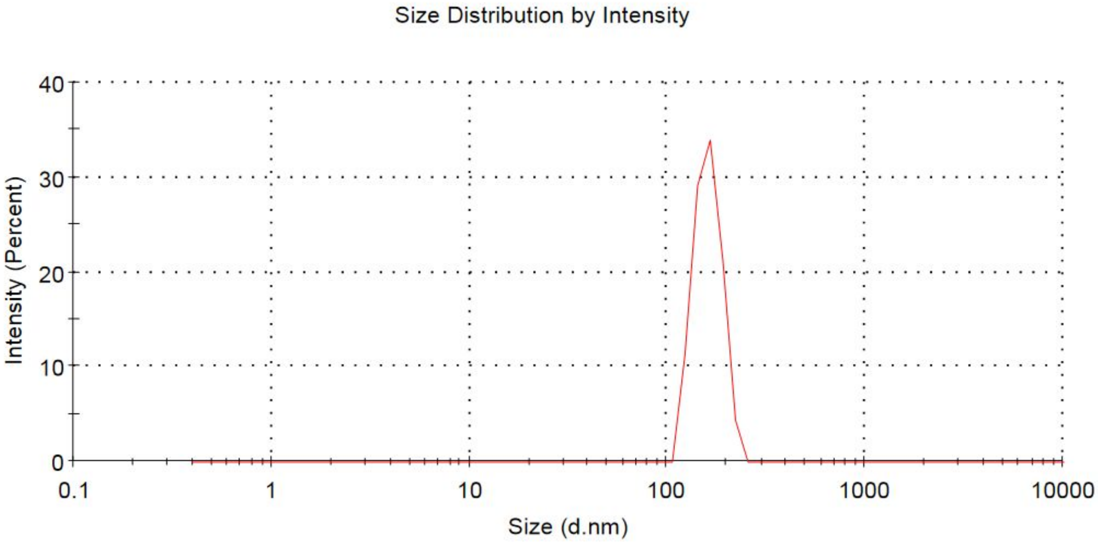

Figure S11. DLS report of AF2 (Z average: 237 nm).

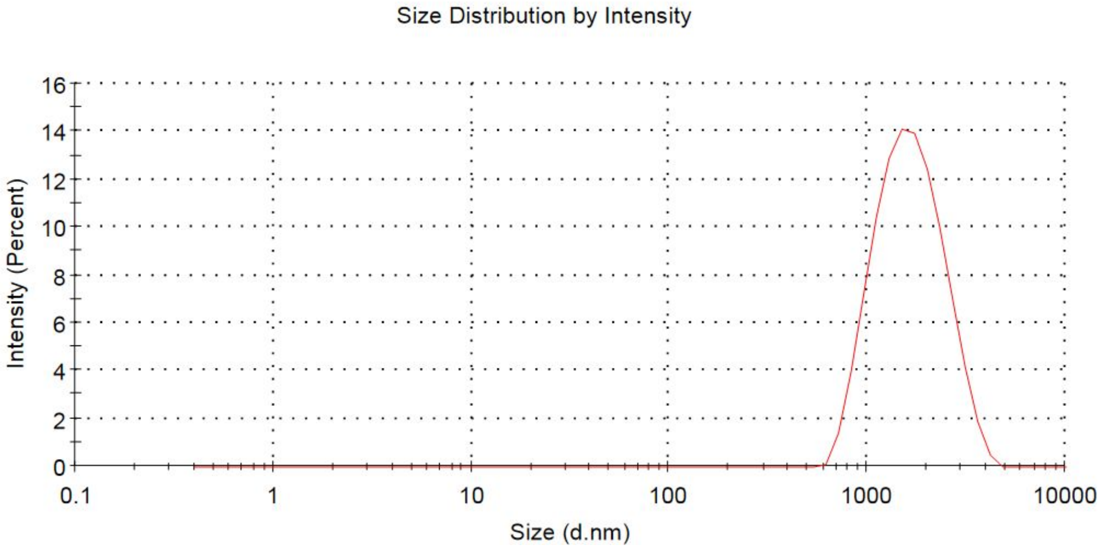

Figure S12. DLS report of AF3 (Z average: 1468 nm).

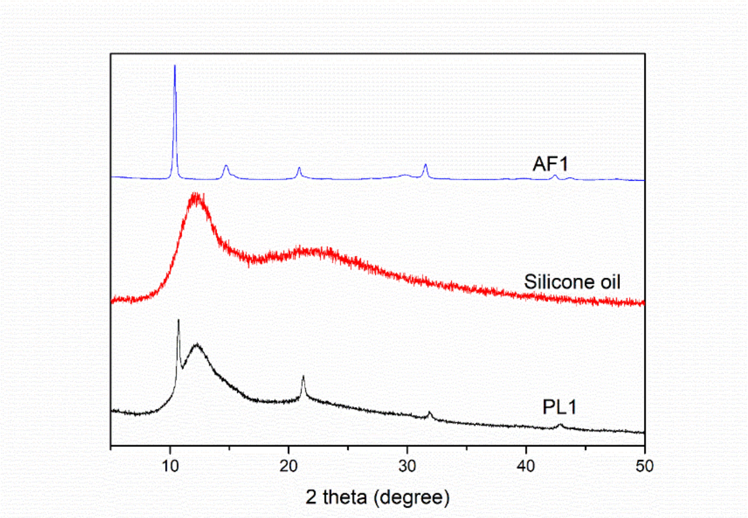

Figure S13. PXRD Pattern of PL1.

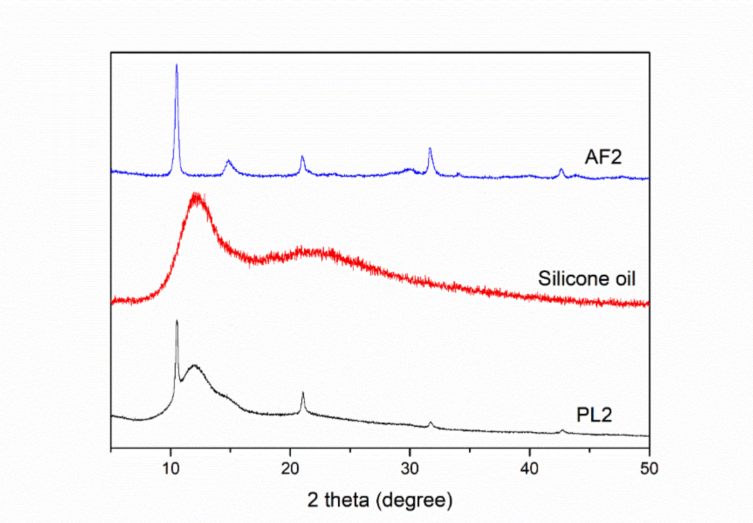

Figure S14. PXRD Pattern of PL2.

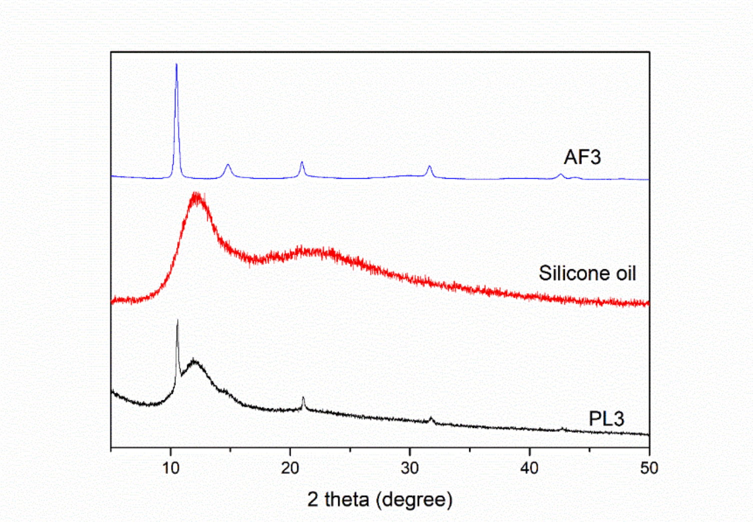

Figure S15. PXRD Pattern of PL3.

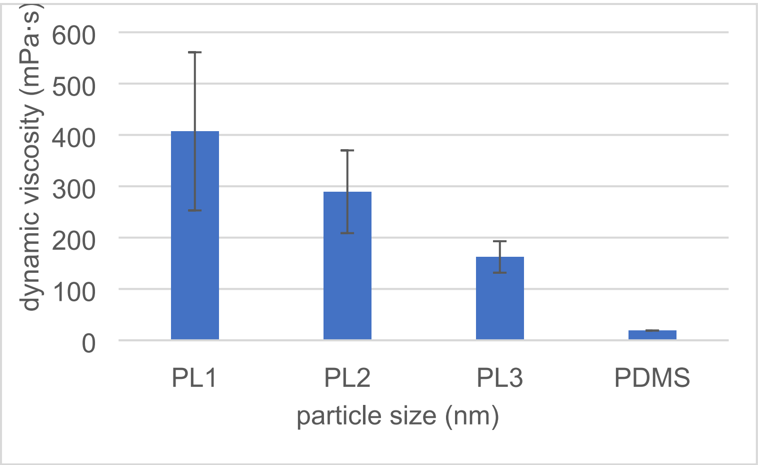

Figure S16. Dynamic viscosities (at 20 °C) of PL1-3 and PDMS.

3. CO_2 uptake measurement

3.1 CO_2 uptake capacity measurement

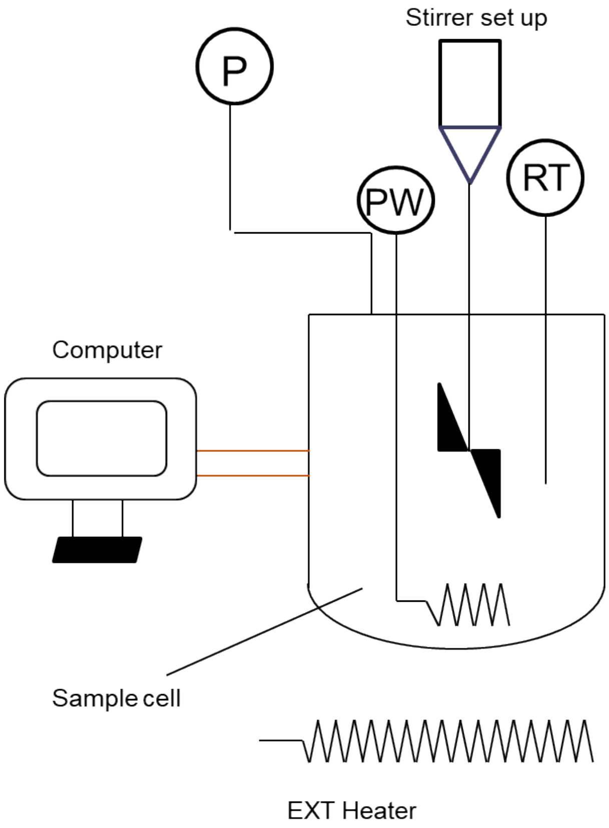

Figure S17. Apparatus for Parr reactor.

3.2 CO_2 uptake kinetics raw data of PL1-2

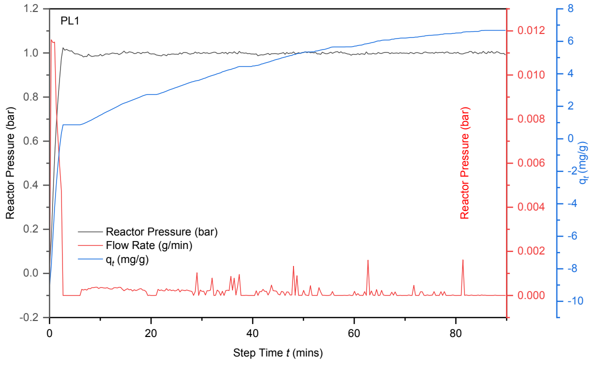

Figure S18. CO_2 uptake versus time for PL1.

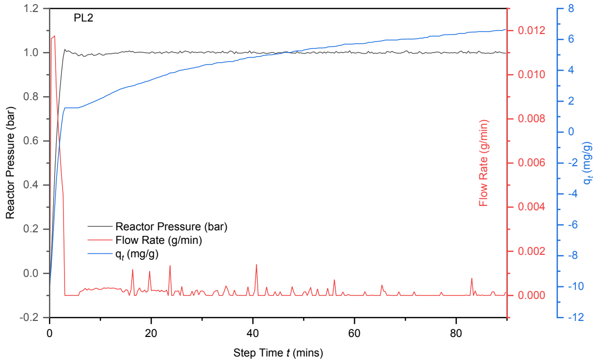

Figure S19. CO_2 uptake versus time for PL2.

4. **Regeneration study**

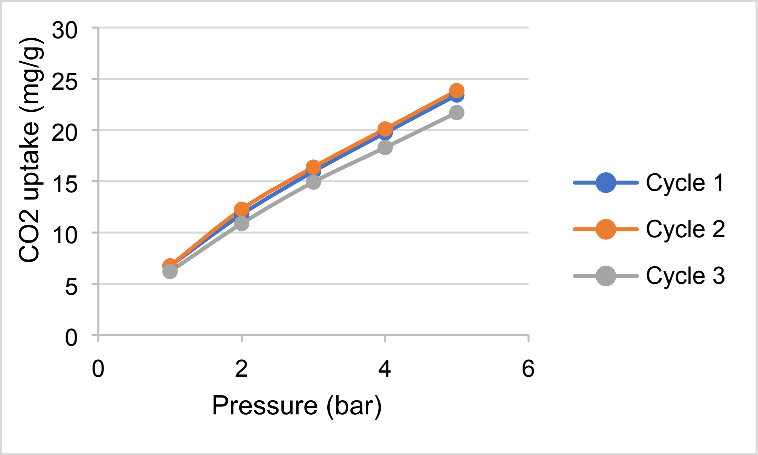

Figure S20. CO_2 uptake cycle measurement for PL1 (1-5 bar).

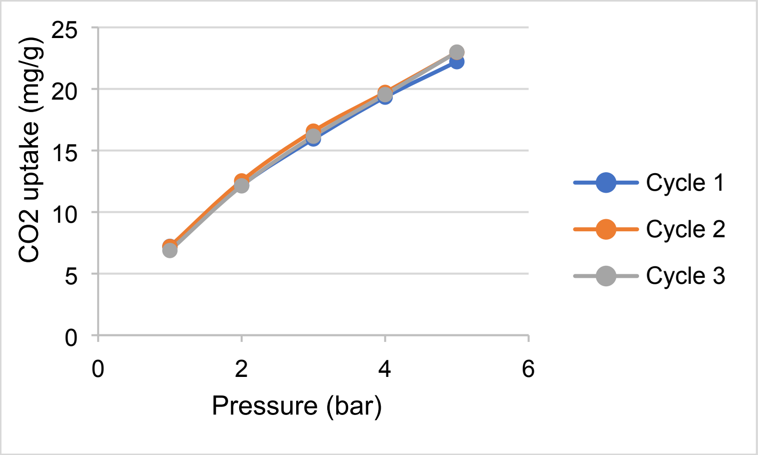

Figure S21. CO_2 uptake cycle measurement for PL2 (1-5 bar).

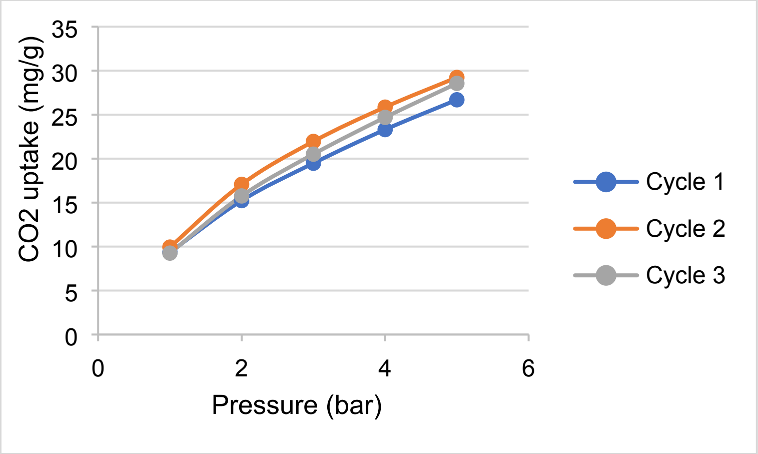

Figure S22. CO_2 uptake cycle measurement for PL3 (1-5 bar).

Stability analysis

4.1 Chemical stability

The chemical stability of porous liquids was determined by PXRD.

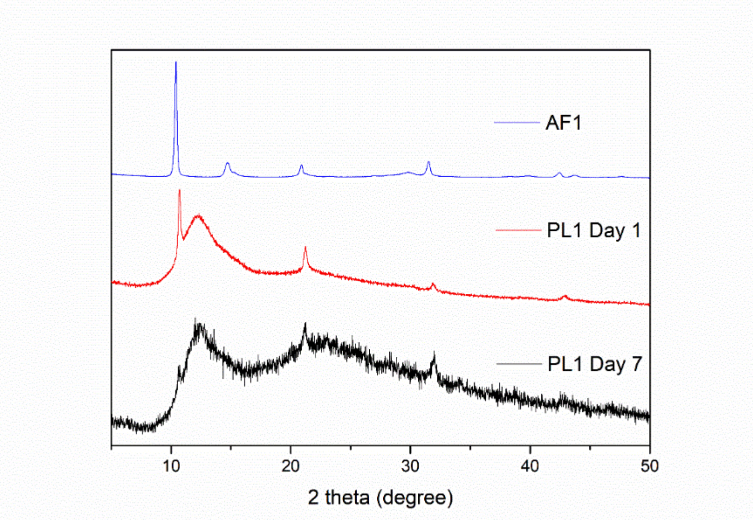

Figure S23. PXRD Patterns of PL1 on 1st and 7th day.

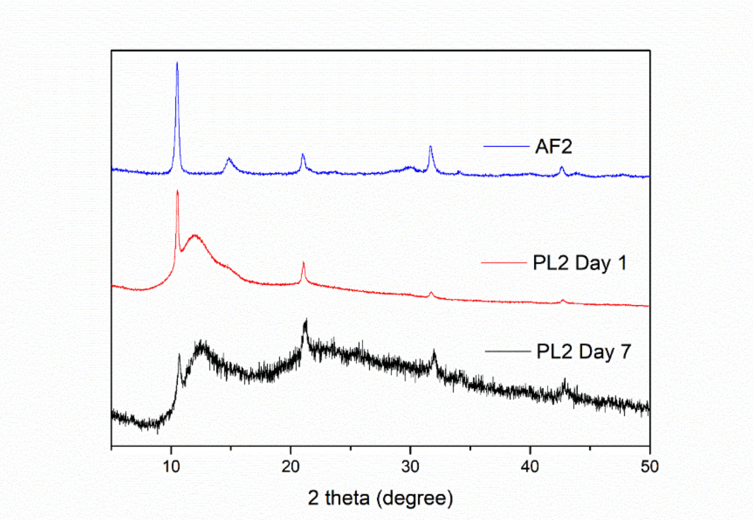

Figure S24. PXRD Patterns of PL2 on 1st and 7th day.

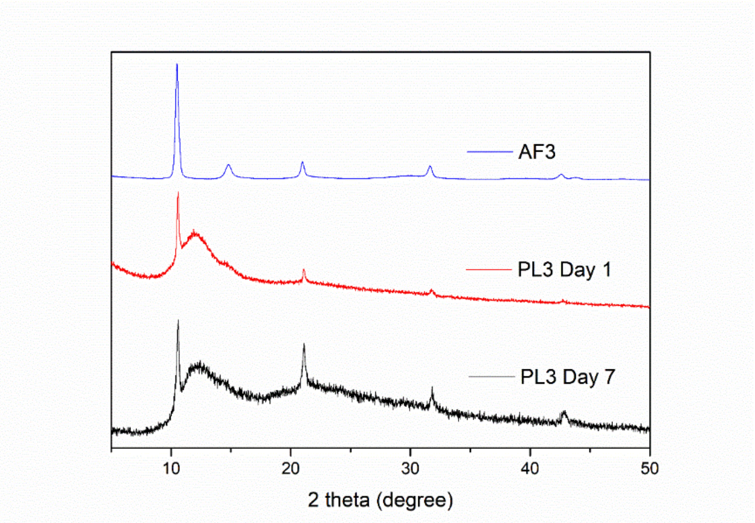

Figure S25. PXRD Patterns of PL3 on 1st and 7th day.

4.2 Sedimentation stability

The sedimentation stability of porous liquids was determined by LumiSizer. Samples were centrifuged at 1500 rpm for 3000 seconds with transmitted light collected every 5 seconds (600 light transmission profiles).

The relative centrifugal force (G-force) can be calculated using the equation below. Therefore, this dispersion analysis is equivalent to ca. 11 days at the standard gravitational force (1 g).

$$\text{G-force (RCF)} = 1.118 \times 10^{-5} \times r \times (\text{rpm})^2$$

G-force = relative centrifuge force (RCF)

r= rotational radius (cm)

rpm= revolutions per minute

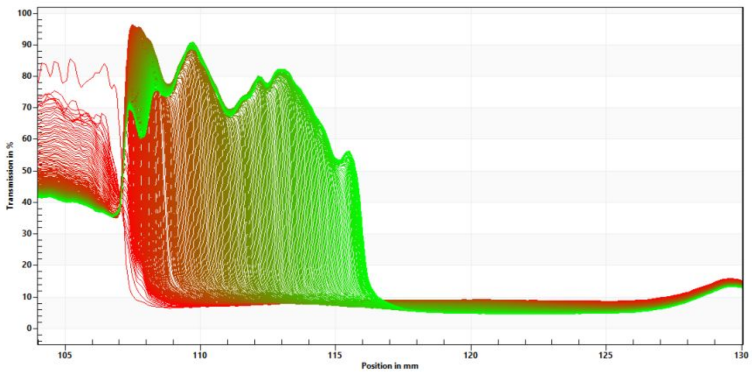

Figure S26. Transmission profile of PL1 obtained under centrifugation at 1500 rpm with 870 nm light.

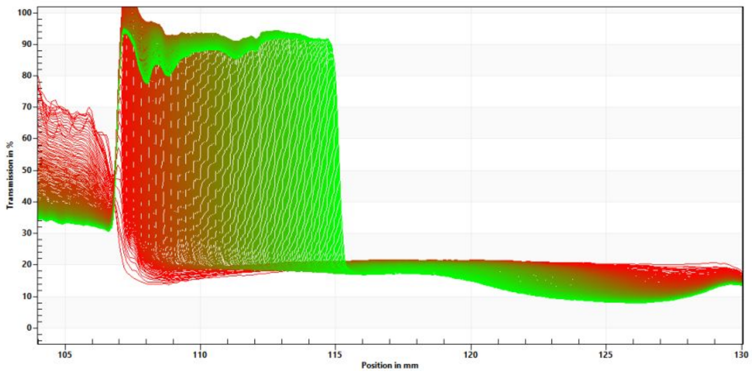

Figure S27. Transmission profile of PL2 obtained under centrifugation at 1500 rpm with 870 nm light.

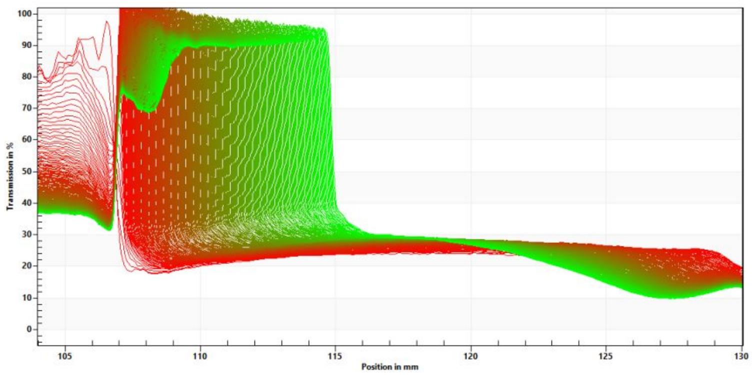

Figure S28. Transmission profile of PL3 obtained under centrifugation at 1500 rpm with 870 nm light.

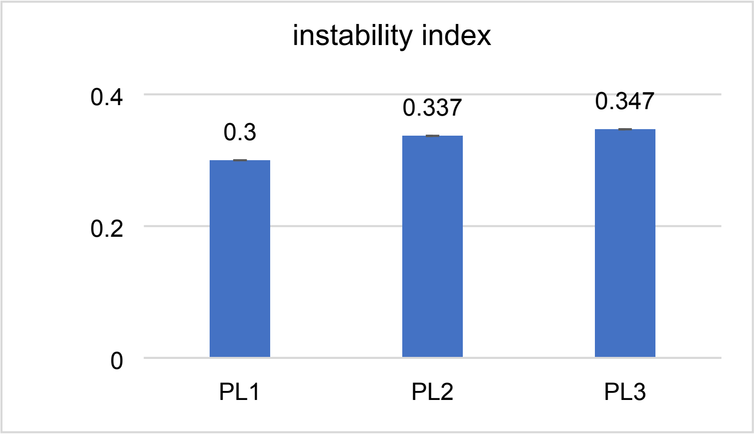

Figure S29. Instability indices of PL1-3.

According to Stokes' law,[1] terminal velocity v increases with enlarging particle diameter R and decreasing viscosity μ because of the balance between weight and buoyancy. Particles with lower terminal velocity tend to disperse in the PDMS instead of sedimentation.

$$v = \frac{2(\rho_P - \rho_f)}{9\mu} g R^2$$

[1] International edition university physics||Newton's laws P115
